# Navigating the complexities of hallux valgus surgery

**DOI:** 10.1007/s00402-024-05607-9

**Published:** 2025-01-23

**Authors:** Sarah Ettinger, Fabian T. Spindler, Sebastian F. Baumbach

**Affiliations:** 1 University Hospital for Orthopaedics and Trauma Surgery, Pius-Hospital Oldenburg, Oldenburg, Germany; 2 Department of Orthopaedics and Trauma Surgery, Musculoskeletal University Center Munich (MUM), University Hospital, LMU Munich, Munich, Germany

Editorial

Surgical hallux valgus (HV) correction is a high-volume orthopedic procedure. Surgical options range from minimally invasive procedures over complex osteotomies to joint fusions. Overall, more than 100 different techniques have been described. For decades the prevailing doctrine has been that different surgical techniques have varying correction potential. Thus, the degree of the deformity is decisive of the surgical technique applied. Consequently, the classification system is an essential cornerstone in the surgical treatment algorithm. However, the currently established classification systems are inconsistent. Referring to the afore-mentioned doctrine this means that techniques like the Chevron osteotomy are more suitable for mild to moderate deformities, whereas the Scarf osteotomy is recommended in moderate and the Lapidus procedure in severe deformities. As is often the case in foot and ankle surgery discussions on which surgical technique is most appropriate were rather eminence- than evidence-based, most likely due to a missing consensus.

For the revision of the German hallux valgus guideline, we took on the daunting task of systematically reviewing the literature on hallux valgus surgery from the implementation of the first German hallux valgus guideline in 2012 to today. The guideline is developed under the patronage of the German Association of Foot and Ankle Surgery (D.A.F.; https://daf-online.de) and published by the German Association of the Scientific Medical Societies (AWMF; https://www.awmf.org), the official registry for all German guidelines.

The aim was to address the primary points of discussion i.e., which surgical technique is suitable for which deformity, what classification should be used and can we define a best-evidence set of outcome scores. Eligible were studies with a moderate to high study quality which compared either two different surgical interventions or the same surgical intervention but for different hallux valgus severities.

In conclusion, the three keystone papers presented in this issue serve as the basis for the newly revised hallux valgus guideline. The first paper focuses on the osseous corrective potential of common open and minimally invasive techniques, whilst the second paper reevaluates the currently used classification systems and in the third paper the authors try to define a best-evidence set of PROMs for hallux valgus surgery. Together these studies provide an evidence-based framework for hallux valgus treatment.

## Data Availability

No datasets were generated or analysed during the current study.

